# Does age in months influence Chinese Holstein dairy cow production performance after controlling for days in milk?

**DOI:** 10.3389/fvets.2025.1743799

**Published:** 2026-01-23

**Authors:** Lei Zhang, Cheng-Long Luo, Qi Mu, Jia-Cheng Liu, Chun-Fang Li, Ya-Bin Ma, Qi Su, Jun-Peng Zhang, Jian-Tao Li, Shu-Yi Zhang, Jian-Min Chai, Yao-Lu Zhao

**Affiliations:** 1 College of Animal Science and Veterinary Medicine, Shenyang Agricultural University, Shenyang, China; 2 College of Animal Science and Technology, China Agricultural University, Beijing, China; 3 Hebei Livestock Breeding Station, Shijiazhuang, China; 4 Ministry of Education Joint International Research Laboratory of Animal Health and Food Safety, College of Veterinary Medicine, Nanjing Agricultural University, Nanjing, China; 5 Guangdong Provincial Key Laboratory of Animal Molecular Design and Precise Breeding, School of Animal Science and Technology, Foshan University, Foshan, China

**Keywords:** analysis of covariance, feed intake, milk yield, PCA, weighted linear model

## Abstract

This study aimed to assess the impact of cows' age (in months) on production performance and evaluate the feasibility of using on-site data to inform precision agricultural practices on farms. Cows were randomly categorized by age in months, and the influence of lactation days was controlled using analysis of covariance (ANCOVA). Weighted linear models (WLM) was employed to control the effect of aggregating data. Principal component analysis (PCA) was applied to explore the relationships among production traits and to elucidate their underlying principles, providing insights for precision management in large-scale farms. The findings were as follows: (1) the age in months had no significant effect on dry matter intake (DMI), residual feed intake (RFI), or daily milk yield (MY; *p* > 0.05); (2) PCA scatter plots revealed no distinct separation between groups, supporting the ANCOVA result that production traits did not differ significantly across the groups. However, the centroid of Group A showed separation from other groups along the second principal component, which was predominantly influenced by age in months, indicating that group distribution in the loading diagram corresponded to the fundamental grouping principle. This study concluded that, at least within the age range of 38.78–53.83 months, cows' age in months did not significantly affect performance. The alignment with ANCOVA results suggests that on-site field data, when analyzed appropriately, can serve as a valuable reference for precision farm management practices.

## 1 Introduction

Milk contains various essential nutrients, including fat, protein, phosphopeptides, and vitamins (1), which play critical roles in human cells and enzyme functions (2). In recent years, many developed nations have shifted their focus from solely increasing cow numbers to enhancing the production performance and efficiency of dairy cows (3). Numerous factors influence dairy cow performance, such as genetic, environmental, and physiological variables (4, 5). Holstein cows are known for their high milk yield and extended peak lactation periods, making them a globally prevalent breed. As cows' physiological states change, significant variations in metabolic levels and production performance occur (6). For instance, first-parity cows, still in a developmental stage, gradually increase milk production, which then declines with age as the body weakens.

To the best of our knowledge, most of the nutritional literature have studied and reported based on data from small-scale scientific trials, and only a small part of the literature is based on large-scale production data from commercial farm and mostly focus on health issue (7). Proper analysis of these production data can offer valuable insights, enabling farm managers to advance precision farming practices and foster the growth of the dairy industry. While numerous studies have investigated the effects of cow parity on production performance (8–10), research on the impact of cows' age in months remains scarce, leaving a gap in specific management guidelines for dairy farms. Most published studies focus on the effect of calving month, such as its influence on lactation curve parameters (11), rather than on the age in months of the animals.

Furthermore, lactating cows typically reach their peak milk production around 50–60 days postpartum, after which production gradually declines, forming the lactation curve (12). In studies, failure to account for the effect of days in milk (DIM) can compromise the accuracy of milk production results, especially in nutritional research (13, 14).

However, limited studies have thoroughly examined the effect of DIM on production performance. Most experiments have controlled for this by selecting animals with similar lactation days, which becomes challenging when there is considerable variation in DIM across field groups. This issue can be addressed by incorporating lactation days as a covariate in the model (15). Therefore, it is crucial to refine the parity effect at the age in months level to better understand how age in months influences dairy cow production performance. This approach, utilizing DIM as a covariate, allows for a more precise analysis based on the farm's specific conditions, offering a theoretical foundation for farmers to understand both population-level and individual-level performance dynamics.

Principal component analysis (PCA) is a dimensionality reduction technique that generates independent composite indicators capable of explaining related variables. The method enables the identification of patterns in the data, which can be visualized in loading diagrams to highlight the relationships among observations and variables (16). PCA, in this context, can be used to examine the distribution and associations of individual cows and herds. Additionally, PCA results can complement the findings from analysis of covariance (ANCOVA). In this study, cows were randomly grouped according to their average age in months, and the effect of lactation days was controlled through ANCOVA to assess the impact of age in months on dry matter intake (DMI), residual feed intake (RFI), and daily milk yield (MY). Furthermore, PCA was employed to explore the relationships between production traits across different age in months groups based on field data from the farms.

The primary aim of this study was to investigate the influence of age in months on the production performance of lactating cows, particularly excluding the effect of DIM. Additionally, the study explored the interrelationships among productive traits while considering age in months, offering insights for enhancing dairy cow feeding management and supporting the feasibility of implementing precision farming practices in large-scale dairy operations.

## 2 Materials and methods

### 2.1 Animals and management

This experiment was conducted in accordance with the Guide for the Care and Use of Laboratory Animals, and received approval from the Animal Ethics and Welfare Committee at Shenyang Agricultural University (22030604). The data were collected from a dairy farm (123°E, 43°N) near Shenyang City in December 2021 to February 2022. The animals used were Chinese Holstein cows, with a total of 2,247 cows included in the data collection, encompassing cows with days in milk ranging from 1 to 305, as well as dry cows. Based on the farm management guideline, the cows were categorized into four groups: “High-yield,” “Mid-yield,” “Low-yield,” and “Dry-cows” and reared in the farm. These groups were housed in 14 zones, each with an average of 161 cows (with a standard deviation of 30 per zone). As the cows were reared in a zone unit, some of the data were observed at multiple animals level, such as the parity and the performance traits, thus some of the statistical data were presented at an average of a sub-group level but not in specific to individual level (e.g., Table 1). The cows were fed a same total mixed ration (TMR), with feeding occurring three times daily and free access to water provided. Milking took place three times a day: morning, midday, and evening. To ensure a relatively balanced sample (i.e., sub-groups) size across groups while capturing potential effects of age on the outcomes, the entire population data was assigned into three groups based on their age in months: 25–44 months (Group A), 45–51 months (Group B), and 52 months and older (Group C). In summary, in the absence of established cut-off points in the literature, the grouping was guided by the distribution of age values as well as the sub-group number in the dataset.

**Table 1:** Statistical description of the animal production performance.

**Traits**	**Age in months group**			**Skewness**	**Kurtosis**
	**A**	**B**	**C**		
*N* (sub-groups in each group)	5	5	4	–	–
Averaged days in milk	124 ± 84.29	195.80 ± 135.23	180.50 ± 137.94	0.11	−1.5
Averaged age in months	38.78 ± 8.83	48.50 ± 2.7	53.83 ± 0.9	−1.31	1.5
Parity	2 ± 0.2	2.18 ± 0.11	2.40 ± 0.29	0.54	1.27
Milk yield by group (kg)	24.38 ± 16.29	23.77 ± 6.47	19.75 ± 7.96	−0.57	−1.33
Averaged dry matter intake (kg/d)	17.42 ± 7.30	17.48 ± 4.55	16.05 ± 3.81	−0.68	−0.68
Averaged residual feed intake (kg)	−2.35 ± 0.44	−1.79 ± 0.56	−1.49 ± 1.37	−0.55	−0.63

The parity and the performance traits were calculated from a zone unit (i.e., multiple animals) level in each sub-group; The data on daily milk production for the herd excludes cows in the dry period.

### 2.2 Data collection

During the research period, data were collected regularly according to the farm's routine procedures, including daily recording of feed intake and residual feed, as well as TMR sample collection for dry matter intake calculation. Due to farm conditions, individual cow feed intake could not be precisely measured, so the average feed intake per zone within each experimental group was used for analysis. Milk production and lactation days were recorded daily using the farm management software associated with the milking equipment. Due to farm constraints, the average weekly milk yield per zone was used for analyzing milk yield performance in this study.

### 2.3 Data analysis

After the experimental data were recorded in Excel (2010 version), data cleaning was performed using a threshold (μ ± 3^*^SD) to exclude outliers from a statistical perspective for further analysis (17). Kurtosis and skewness were used to assess the normality of the data. A greater kurtosis value indicates steeper data distribution, while a lower value suggests a flatter distribution. Similarly, a higher absolute value of skewness signifies greater data skewness. Data were considered normally distributed if the absolute values of both kurtosis and skewness were less than 2 (18). According to the kurtosis and skewness results presented in Table 1, all data were found to follow a normal distribution.

Due to the large diversity of days in milk in each treatment group and the large gap between groups, when analyzing the effect of the age in months on feed intake (DMI), residual feed intake (RFI) and daily milk yield (MY), it might be affected by DIM. To address this, the analysis of covariance (ANCOVA) method was employed to eliminate the influence of DIM, allowing for a more accurate evaluation of the effects of age in months on DMI, RFI, and MY, thereby improving the quality of the analysis. Since age in months provides a more specific measure of parity in production, and a positive correlation between parity and age in months was observed in this study (*r* = 0.71, *p* < 0.05), the average parity in each group was approximately 2. Therefore, the effect of parity was not considered. However, since the lactation and feed intake curves are non-linear from 50 to 60 days postpartum, which violated the model's assumption of linearity, records with a DIM of less than 50 days were excluded to improve the accuracy of the analysis. Additionally, one record from each group was excluded from the MY analysis due to its occurrence during the dry milk period.

Descriptive statistics and correlation calculations were performed using the base package of R 4.1.3 software. ANCOVA was conducted using the rstatix package, with DIM as a covariate, age in months as the independent variable, and DMI, RFI, or MY as dependent variables. Factors such as management levels were included as random errors. Traits exhibiting significant differences were reported as least squares means after accounting for the effects of covariates. Prior to conducting the ANCOVA, tests for homogeneity of variance, linearity between covariates and dependent variables, and a slope consistency hypothesis test among groups were performed. A significance level of 0.05 was set for all statistical tests. The model used in the ANCOVA analyses was as follows:


$$\begin{array}{l} yij=\mu \;+\;Ti\;+\;\beta Xij\;+\;\varepsilon ij \end{array}$$ (1)

where yij represents the jth observation of the dependent variable for the ith group; μ is the overall mean; Ti denotes the treatment effect for the ith group; Xij is the corresponding covariate; β is the slope of the linear regression of the covariate corresponding to the dependent variable; and εij is the random error. To further validate the results from the ANCOVA analysis, the individual production indicators discussed earlier were used as input variables. After standardization, PCA was conducted. A scree plot was employed to determine the number of principal components to retain, using a threshold value of 1 (19). Graphs depicting the distribution of variables, groups, and individuals were created to observe how individuals were distributed across different month-age groups. Specifically, individual samples were projected onto the principal component space, with colors indicating group membership. For each group, a centroid was calculated as the arithmetic mean of the individuals' coordinates on the first two principal components, representing the group's central tendency in the first two dimensions. These centroids were plotted as group-level mean points. To visualize within-group variability and uncertainty, 95% confidence ellipses were drawn around each group, assuming multivariate normality. In brief, each ellipse was constructed using the empirical covariance matrix of the group's individuals and the chi-square distribution, forming a boundary expected to enclose approximately 95% of future observations. The FactoMineR and factoextra packages of R 4.1.3 software were utilized for conducting the PCA and visualizing the result.

In order to control the risk of ecological fallacy induced by data aggregation, the weighted linear model (WLM) was employed (20–22). In brief, the WLM could account for unequal group sizes, using group-level means as outcomes and group-specific sample sizes as weights in the model. This way of doing could improve estimation precision and is suitable when individual-level data is unavailable. Moreover, by incorporating covariates and weighting by group sizes, WLM helps mitigate ecological bias arising from the use of aggregated data.

## 3 Results

**Figure 1:**
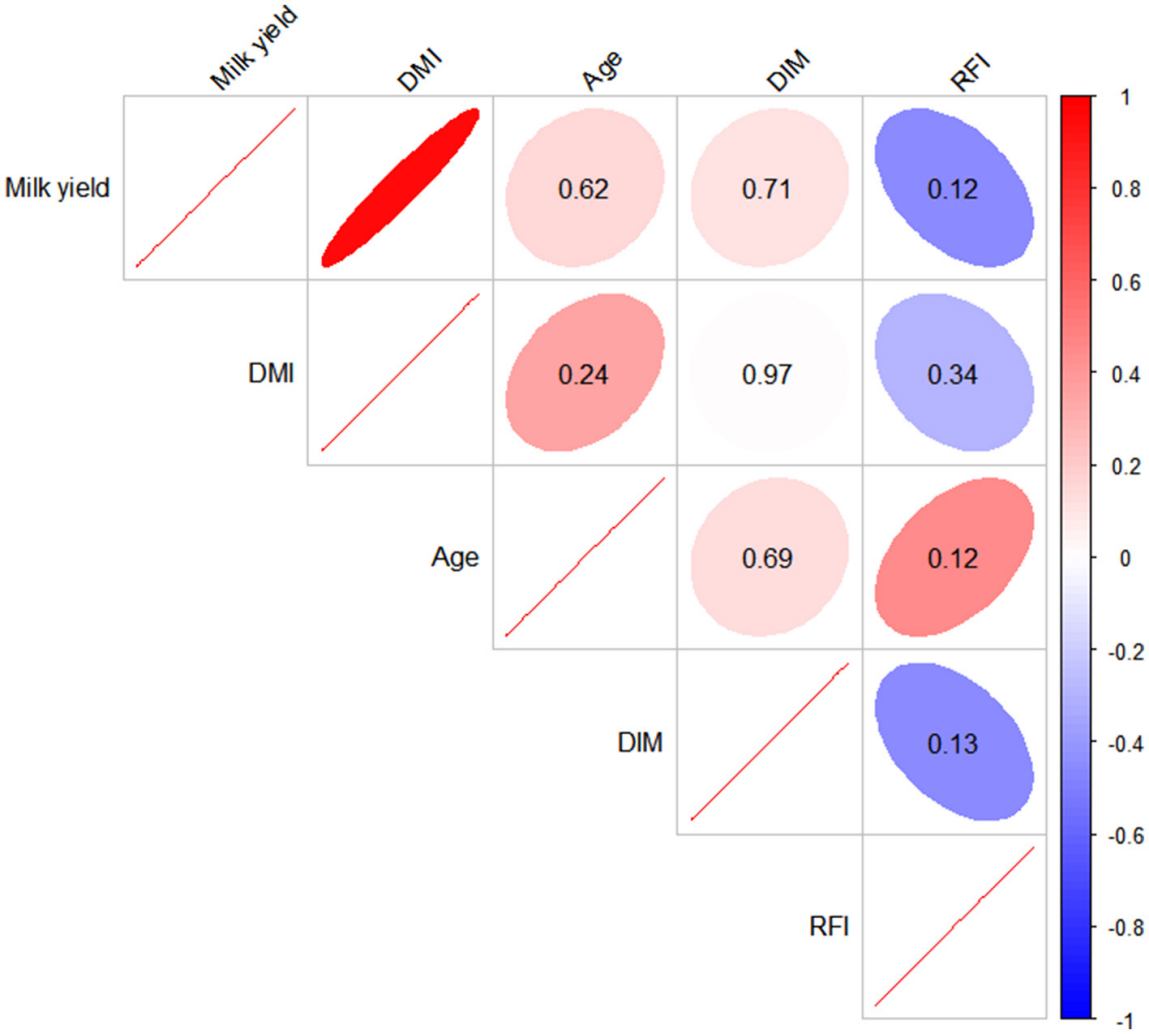
The Pearson correlation among milk yield, dry matter intake (DMI), age in months (age), days in milk (DIM), and residual feed intake (RFI). (Color in red represents positive correlation, blue indicates negative correlation; the number in ellipses means *p* value (ellipses without number inside denotes *p* value less than 0.05).

After cleaning the data using the criterion μ ± 3 ^*^ SD, no records were excluded. Therefore, the final analysis was based on 13 average data points, representing the average data for each of the 14 rearing zones (excluding one data point with DIM of less than 50 days). The results of the skewness and kurtosis analyses, presented in Table 1, indicated that the data followed a normal distribution. As presented in Table 1, the mean number of cows in each group were 143 (averaged from five sub-groups), 177 (averaged from five sub-groups), and 163 (averaged from four sub-groups), with standard deviations ranging from 10 to 46, respectively. The mean DIM for the groups were 124.00, 195.80, and 180.50 days, with standard deviations ranging between 84.29 and 137.94. The mean daily milk yield ranged between 19.75 and 24.38 kg, with standard deviations between 7.96 and 16.29 kg. Mean DMI was 17.42, 17.48, and 16.05 kg, with standard deviations ranging from 3.81 to 7.30 kg. Mean age in months was 38.78, 48.50, and 53.83 months for the groups, with standard deviations ranging from 0.90 to 8.83 months. Mean RFI was −2.35, −1.79, and −1.49 kg for the groups, with standard deviations of 0.44, 0.56, and 1.37 kg. The kurtosis and skewness values for each trait ranged from −1.41 to 0.54 and −1.50 to 1.84, respectively. The Pearson correlation information is given in Figure 1. As observed, all the *p* values of the correlation were higher than 0.05, except for the correlation coefficient between milk yield and DMI. Age in months had positive correlation coefficient with other productive traits but not significant (Figure 1). In specific, the correlation coefficient value ranged from 0.15 to 0.46.

### 3.1 Effect of age in months of cows on feed intake

**Figure 2:**
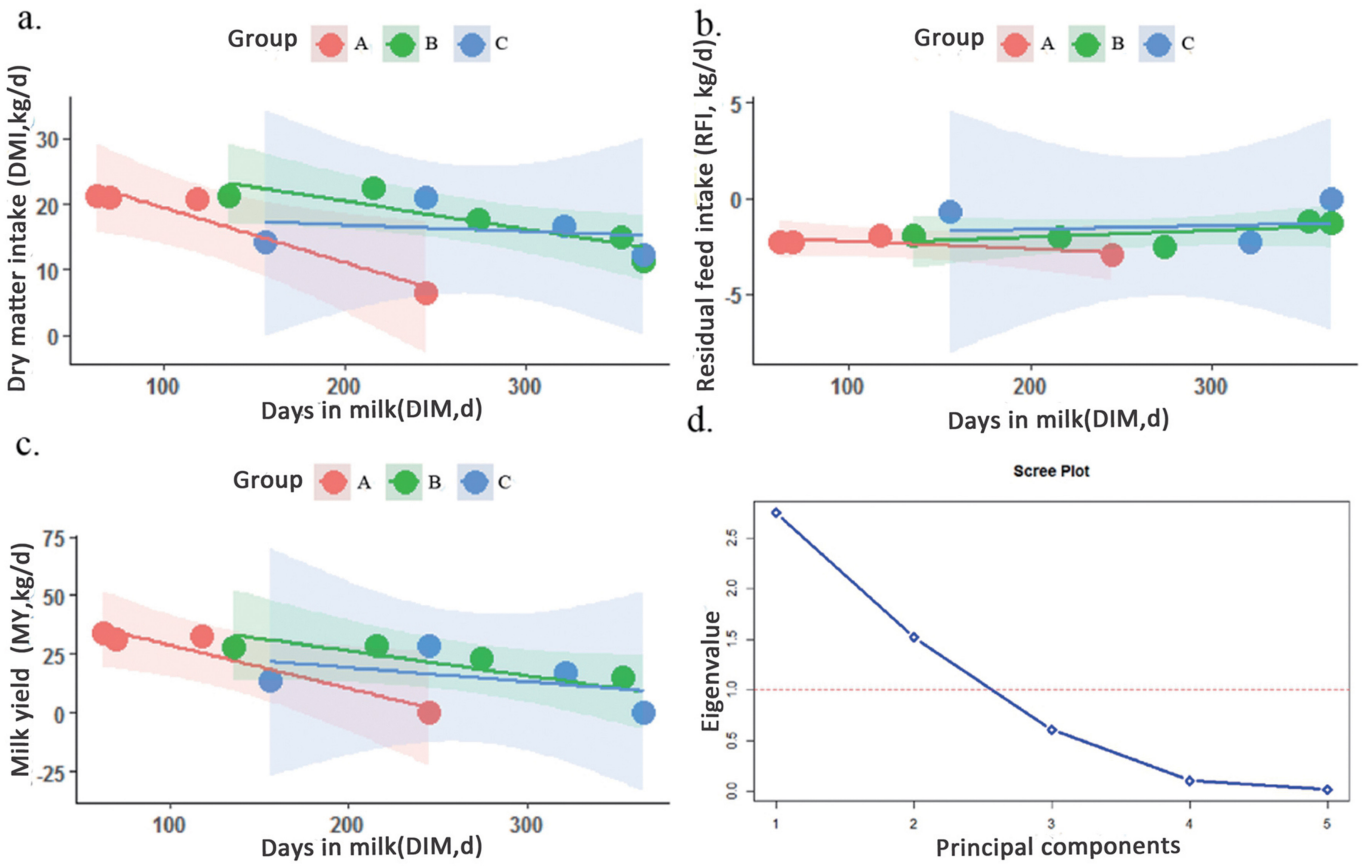
The linear relationship between the covariate days in milk (DIM) and each dependent variable: **(a)** dry matter intake (DMI), **(b)** residual feed intake (RFI), **(c)** milk yield (MY), and **(d)** the principal component analysis scree plot. (Color in red represents group A, green indicates group B, and blue means group C; the dash red line in plot d means the threshold at 1.0 for the scree plot).

The variance homogeneity test yielded a *p*-value of 0.52, and the regression residual normality test yielded a *p*-value of 0.09. The parallelism of the regression slopes across groups was nearly identical, with the slopes of DMI and DIM ranging from −0.08 to −0.01 and an *R*^2^ value of up to 0.59 (Figure 2a), meeting the fundamental requirements for the analysis of covariance.

ANCOVA was used to assess the effect of age in months on DMI, yielding a *p*-value of 0.36 (Table 2). This indicated no significant difference in DMI between the different age in months groups after correcting for the covariate DIM. The least squares means of DMI for each group are presented in Table 2. From these results, it can be concluded that the difference in DMI between groups A, B, and C ranged from 1.30 to 6.30 kg, but the differences were not statistically significant (*p* > 0.05), suggesting that age in months did not have a significant effect on the DMI of lactating cows.

**Table 2:** Least squares means of production performance of each age in months group estimated by different models.

**Modeling methods**	**Traits**	**Age in months groups (LSMean** ±**SE)**			***p*-Value**
		**A (*****N*** **=** **5)**	**B (*****N*** **=** **5)**	**C (*****N*** **=** **4)**	
Analysis of covariance	Dry matter intake (kg)	13.10 ± 2.33	19.40 ± 1.8	18.10 ± 2.01	0.36
	Residual feed intake (kg)	−2.23 ± 0.55	−1.84 ± 0.43	−1.54 ± 0.48	0.87
	Daily milk yield (kg/d)	29.00 ± 4.78	25.30 ± 3.33	21.20 ± 3.71	0.38
Weighted linear model	Dry matter intake (kg)	17.40 ± 2.50	17.50 ± 2.44	16.00 ± 2.69	0.80
	Residual feed intake (kg)	−2.73 ± 0.37	−1.66 ± 0.43	−1.41 ± 0.40	0.17
	Daily milk yield (kg/d)	29.10 ± 4.70	25.20 ± 3.03	21.00 ± 3.50	0.09

The N values for each group of milk production, excluding the dry period data, are 3, 4, and 3, respectively; The number of animals involved in each group was 143, 177, and 163 for A, B, and C group, respectively.

The results of WLM are presented in Table 2 (lower part). In summary, the LSmeans among groups were not differed (p > 0.05). The LSmeans of each group were similar to the ones calculated from ANCOVA except the DMI of A group.

### 3.2 Effect of cows' age in months on RFI

Consistent with the results on the effect of age in months on feed intake, data among groups supported the hypothesis of variance homogeneity (*p* > 0.05), and the residuals from the regression model followed a normal distribution (*p* > 0.05). Additionally, the slopes of the regression lines between groups were parallel, with RFI and DIM slopes ranging from −0.02 to 0.004, close to zero, and the highest *R*^2^ value reaching 0.93 (Figure 2b), meeting the fundamental assumptions for covariance analysis.

As shown in Table 2, the least squares means of RFI for the groups were −2.23, −1.84, and −1.54 kg, with a maximum inter-group difference of 0.69 kg. ANCOVA yielded a *p*-value of 0.87 for the effect of age in months on RFI (Table 2), indicating no significant difference in RFI between the age in months groups.

### 3.3 Effect of age in months of cows on daily milk yield

The effect of age in months on daily milk yield was also assessed. The *p*-values from the variance homogeneity test and the regression residual normality test were 0.14 and 0.66, respectively, both greater than 0.05. Similar to the previous two traits, the parallelism of the slopes of the regression linearity groups of the DIM on daily milk yield was basically the same (the slopes of the milk yield vs. the DIM were spread out between −0.06 and 0.02, which was close to 0, The highest *R*^2^ was 0.83, as shown in Figure 2c), which also met the basic requirements of the analysis of covariance.

As presented in Table 2, the least squares means of daily milk yield across the groups ranged from 21.20 to 29.00 kg, with a maximum difference of 7.80 kg between groups. However, after performing ANCOVA, no significant effect of age in months on milk yield was observed (*p* > 0.05), once the influence of DIM was excluded.

### 3.4 Effect size and 95% confidence intervals of ANCOVA and WLM results

**Figure 3:**
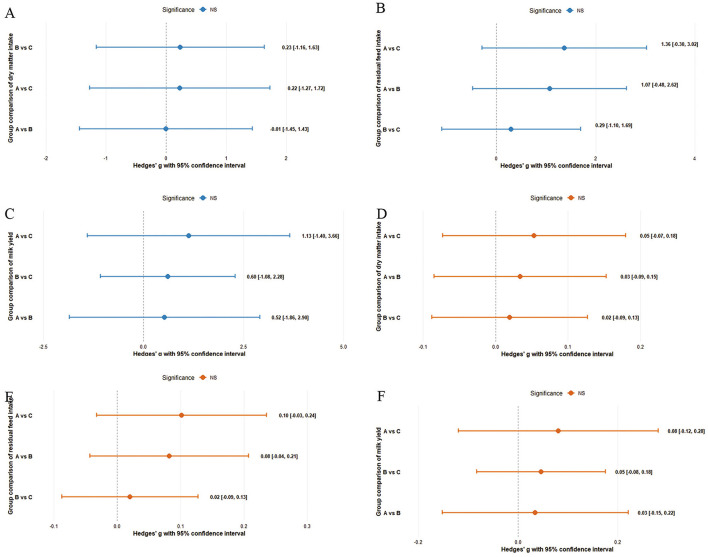
Effect size and 95% confidence intervals of analysis of covariance and weighted linear model results (Color in blue represents analysis of covariance, red indicates weighted linear model; **(A)** and **(D)** denotes dry matter intake; **(B)** and **(E)** indicating residual feed intake; **(C)** and **(F)** means milk yield).

The pairwise effect sizes of each trait as well the corresponding 95% confidence intervals are shown in Figure 3 (*p* > 0.05). As observed, the effect size ranged between −0.01 for DMI and 1.36 for RFI in the ANCOVA (Figures 3A–C). However, the results were not significant due to the 95% confidence intervals covered 0. Similarly, the effect size (Figures 3D–F) of all traits resulted from WLM analysis were between 0.02 for DMI and 0.08 for milk yield. The corresponding 95% confidence intervals also included 0.

### 3.5 Results of principal component analysis

The principal component extraction details from the PCA for each variable are presented in Table 3. The scree plot (Figure 2d) was used to determine the number of principal components to retain, selecting those with eigenvalues greater than 1. Based on the information in Table 3 and Figure 2d, two principal components were extracted, explaining 55.03 and 30.40% of the total variance, respectively. Thus, the cumulative variance explained by these two components was 85.43%.

**Table 3:** Explained variance of each principal component.

**Components**	**Eigenvalue**	**Percentage of explained variance (%)**	**Cumulated percentage of explained variance (%)**
Comp1	2.75	55.03	55.03
Comp2	1.52	30.41	85.44
Comp3	0.61	12.12	97.55
Comp4	0.10	2.09	99.65
Comp5	0.02	0.35	100.00

**Figure 4:**
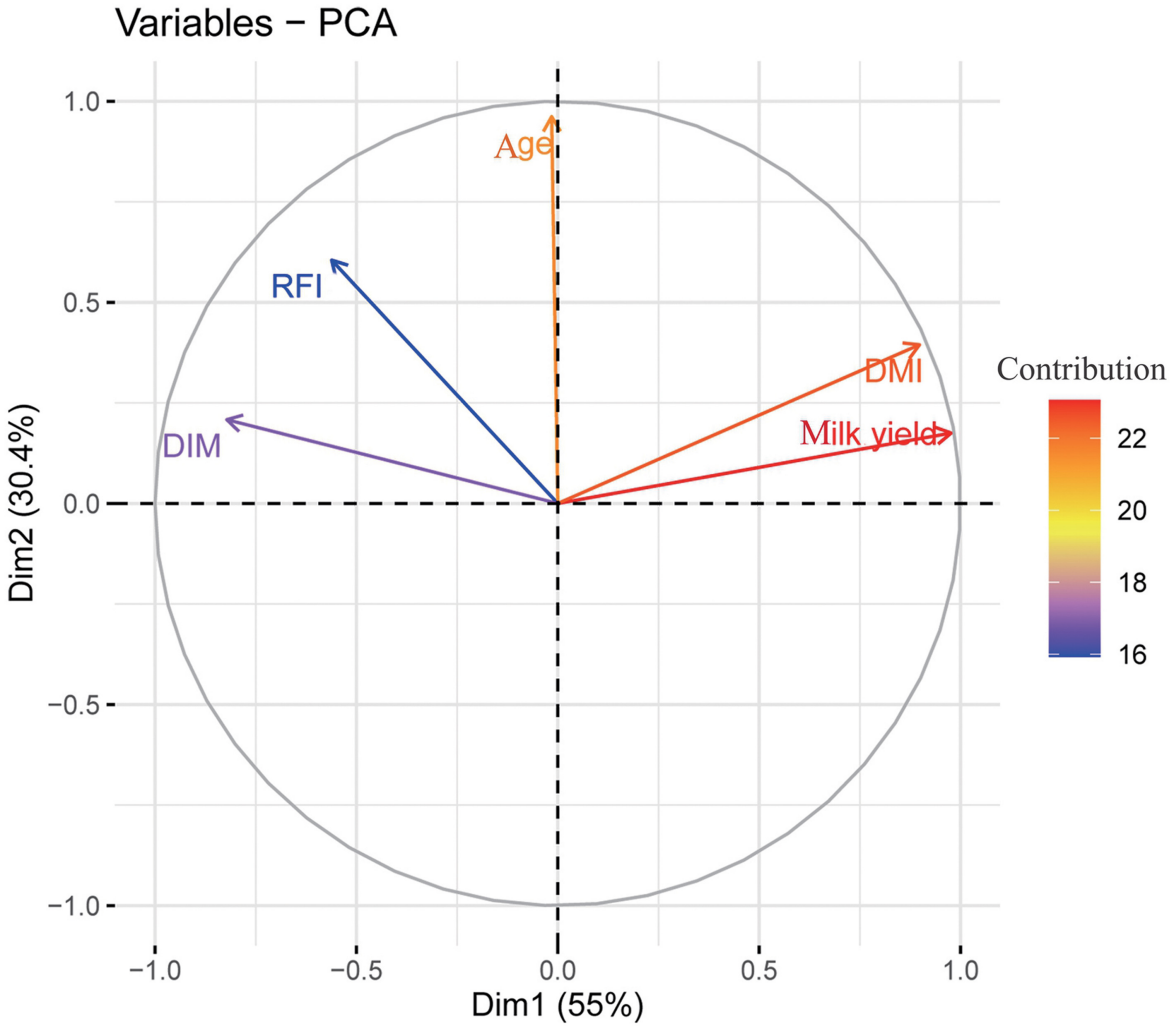
Loading plot of principal component analysis (PCA). (The length and color of each arrow denotes the contribution of the trait to each dimension; color in red means higher contribution and purple denote lower contribution).

Figure 4 illustrates the relationships between the variables, with Dim1 and Dim2 representing the first and second principal components, accounting for 55.03 and 30.40% of the variance, respectively. Dim1 primarily captures the effects of daily milk yield (Milk yield), DMI, days in lactation (DIM), and partial RFI, while Dim2 primarily represents age in months (Age), Milk yield, DMI, and RFI.

**Figure 5:**
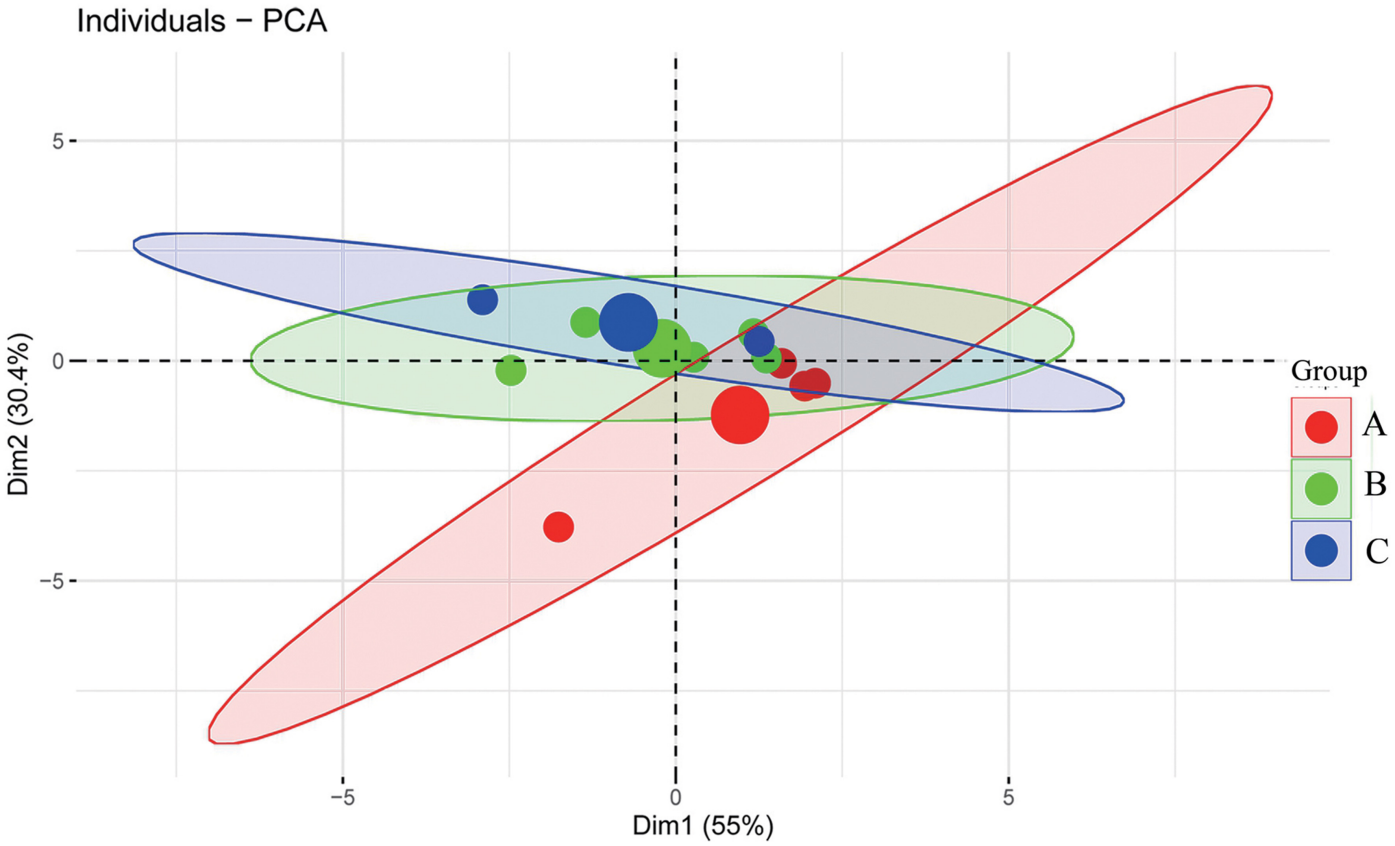
Individual and group distribution plot based on the principal component analysis (PCA) results (the ellipses around each group represent the 95% confidence).

The angles between Milk yield and DMI, as well as between RFI and DIM, are both less than 90°. In contrast, the angles between milk yield or DMI with RFI or DIM are greater than 90°, and the angles between these four variables and age in months are less than 90°. Smaller angles between variable vectors indicate stronger correlations between the variables, while angles approaching 90° suggest weaker correlations. The longer the vector, the greater the loading of that variable in the corresponding principal component direction. The individual scatter plot in two-dimensional space (Figure 5) further demonstrates the ability of PCA to distinguish and display the characteristics of different groups. The distribution of groups across different age in months shows notable separation, with clusters forming within the data. Specifically, the larger dots in each group represent the group centroids, and it is evident that the centroid of group B is closer to that of group C, while being slightly farther from the centroid of group A.

## 4 Discussion

To further validate the findings derived from ANCOVA, we applied a weighted linear model (WLM) to account for potential ecological bias induced by aggregated data and group-level sample size variability. The estimated least squares means obtained from the WLM were numerically consistent with those from the ANCOVA, and the between-group differences remained statistically non-significant. These results suggest that the absence of significant group effects observed in the initial ANCOVA analysis, despite not explicitly addressing ecological fallacy, was not substantially biased. Therefore, the agreement between the two modeling approaches supports the robustness of the current findings and provides a sound basis for subsequent interpretation and discussion.

### 4.1 Effect of age in months on cow feed intake

After data cleaning, no records were excluded, indicating that the data quality from this farm was acceptable and aligned with the management standards typically observed in dairy cow farms in China. The daily milk yield for each group, with DIM ranging from 124.00 to 195.80 days, varied between 19.75 and 24.38 kg, falling within the reasonable performance range for Holstein cows as recommended by the international ICAR standards (23). The DMI ranged from 16.05 to 17.42 kg/d, which was slightly lower than the 20.87 kg/d observed in Holstein cows with an average parity of 2.3, as reported by De Boever et al. (24). This difference may be attributed to the lower productivity and higher intra-group variability at the farm used in this study. However, despite the considerable variability in daily milk production and DMI, the feeding efficiency (i.e., the ratio of milk production to DMI) of the animals in this study ranged from 1.23 to 1.40, which aligns with the recommended range of 1.20–1.80 (25), indicating that the animal population was within a positive production level.

As presented in Table 2, after correcting for the effect of DIM, the DMI values for each group changed. The DMI of Group A decreased from 17.42 kg/d before correction to 13.10 kg/d, while the DMI for the other two groups increased slightly by approximately 2 kg. This suggests that DIM had a more pronounced effect on DMI. After adjusting for DIM, although trends in DMI between groups were observed, such as 6.30 kg higher intake in Group B compared to Group A and 5.00 kg higher intake in Group C compared to Group A, there was no statistically significant effect of month age on the DMI of dairy cows (*p* > 0.05). The corrected feed intake showed a trend of increasing and then decreasing, although not statistically significant. This trend is consistent with that described by Reshalaitihan et al. (26), where changes in feed intake were related to parity, even though the specific focus on age in months was not addressed. This provides a reference for the present study. After 16 months of age, Holstein cows reach body maturity, which may explain the lack of statistical difference in DMI after correcting for DIM. This is supported by the mean ages of 38.78, 48.50, and 53.83 months for the respective groups, suggesting that animals in each group had already reached maturity. Furthermore, the lack of significant differences in performance (e.g., daily milk yield) further explains the absence of a statistically significant difference in DMI.

### 4.2 Effect of age in months of cows on residual feed intake

As shown in Table 2, no significant differences (*p* > 0.05) in RFI were observed between the different age groups, indicating that age in months had no significant impact on RFI after accounting for the effect of DIM. RFI, defined as the difference between actual feed intake and predicted feed intake based on expected production performance, is widely regarded as an indicator of feed efficiency in animals (27). A lower (typically negative) RFI value indicates greater productivity (28). After correcting for DIM, the RFI values remained relatively unchanged, with the smallest value in Group A and the largest in Group C, but no statistical differences were found between groups (*p* > 0.05).

However, there was a tendency, albeit not significant, for RFI to increase with age in months, which aligns with the trend observed by Grandl (29) in their study involving groups with considerable age variation (6–120 months). One potential reason for the lack of statistical significance in this study could be the limited sample size and the diversity of age in months within groups. Increasing the data size in future studies would enhance statistical power, providing results that more closely reflect real-world conditions.

### 4.3 Effect of age in months of cows on daily milk production

Table 2 also reveals that, after correcting for the effect of the covariate DIM, the daily milk yield of the different age in months groups was 29.00, 25.30, and 21.20 kg, respectively. These values changed by 4.62, 1.53, and 1.45 kg, compared to pre-correction values. Numerically, the daily milk yields of Groups A and B, corrected for DIM, were higher than that of Group C, with differences of 7.8 and 4.1 kg, respectively. However, similar to DMI and RFI, no significant effect of age in months on daily milk yield was observed (*p* > 0.05). Given that cows' physiological development varies with age, milk yield typically increases as body functions develop and reach their peak, after which it declines with further age (25). The average parity in each group in this study was the second parity, making the lack of significant differences reasonable. Although the age in months variability within each group was relatively low (overall coefficient of variation, CV = 16%), the lack of significant differences may have been limited by the sample size. Increasing the data size would likely improve the ability to detect age in months effects on dairy cow production performance in future studies.

PCA is a dimensionality reduction algorithm that generates mutually independent composite indicators, called principal components, which explain the relevant variables. This method clusters individuals and variables in loading diagrams to effectively display the data characteristics and reveal the relationships between them (16). In this experiment, PCA was applied to the production performance data of Holstein cows, converting trait variables with different units into uniform linear scores. By simplifying redundant information through dimensionality reduction, differences between groups and individuals were analyzed. As shown in Table 3, the cumulative variance explained by the first two principal components was 85.43%, exceeding the 80% threshold, confirming the representativeness of the loading diagram for these components.

The PCA loading plot (PCA-biplot) illustrates the correlations between component variables and reflects the relationships between the production indices as well as between groups and individuals. As shown in Figures 4, 5, daily milk yield, DMI, RFI, age in months, and DIM—key production indicators for dairy cows—can be reduced to two dimensions, enabling observation of both individual and group distribution based on the principal components, along with the variable loading vectors. For example, daily milk yield and DMI exhibit a strong positive correlation: as one increases, so does the other. This aligns with findings by Zhang et al. using a large group, who described a similar trend in lactating cows, where DMI and milk production evolved together (12). Additionally, it has been reported that as age in months increases, body function development in cows, such as an increase in rumen volume, leads to greater feed intake (12). Figure 4 highlights that feed intake is a critical factor influencing lactation performance in dairy cows, reinforcing the positive correlation between DMI and MY observed in prior studies (30). These results further support the validity of the PCA findings in this study. However, age in months was projected closely to Dim2 which although having acute angle with daily milk yield, DMI, RFI, and DIM, the angle was not close to zero degrees, especially for DIM and daily milk yield. This explained why the difference for those traits were not significant even modeled based on the ANCOVA and WLM algorism as the angle were even reach 90 degrees. Further, the fact that correlation of age with other traits were not significant (Figure 1) also confirmed age in months had no effects on studied productive traits.

Similarly, Figure 5 illustrates that the individual scatters of each group were not clearly distinguishable along the first principal component, suggesting a considerable diversity of samples within each group. However, along the second principal component, the three groups displayed a hierarchical distribution corresponding to their respective age in months. Specifically, the lower age in months in Group A was aligned with the negative direction of Dim2, while the higher age in months in Group C was positioned toward the positive direction of Dim2. Overall, Group A was more concentrated toward the positive direction of the first principal component, indicating higher production performance, while Groups B and C were situated more toward the negative direction, reflecting relatively lower production efficiency. These observations are consistent with the ANCOVA results, which showed that Group A had a lower RFI compared to the other groups, along with a relatively higher milk yield (Table 2).

In summary, based on ANCOVA and the WLM analysis, after correcting for the DIM effect, the DMI, RFI, and MY of the different age in months groups exhibited changes compared to pre-correction values, highlighting the importance of adjusting for DIM. However, despite these corrections, no significant differences in corrected DMI, RFI, or MY were observed across the groups. The ANCOVA results suggest that, at least within the age range of 38.78–53.83 months, age in months does not significantly influence the productive performance of lactating dairy cows. What is more, according to the effect size evaluation from both the ANCOVA and WLM, the age in months effect was small (0.02) to medium (1.36) with 95% confidence intervals across 0 which confirmed the findings above. Additionally, the PCA results revealed clear relationships between performance traits, such as the strong association between DMI and MY with Dim1, emphasizing the role of DMI in lactation performance. The score plot further clarified the distribution of inter-group and inter-individual relations. The lack of clear separation between groups based on age in months served as additional confirmation of the ANCOVA and WLM findings, suggesting that within this age range, age in months is not a major factor influencing the production performance of dairy cows at least at a sub-group level. Due to the on-site limitation, the current research aggregated the individual animals into sub-groups to do the analysis. Although many researches in poultry science and pig science field often adopt the aggregate data as the smallest unit for statistical analysis (i.e., a replicate containing many animals as the smallest unit) (31–33). This increased the risk of Type II errors and preventing individual-level inferences and eliminates valuable within-group variability and raises concerns about ecological fallacy. So, future research could benefit from increasing the sample size (especially the individual level data) or the number of the sub-groups to enhance statistical power and the reliability of conclusions.

## 5 Conclusions

After accounting for the effect of days in milk (DIM) using ANCOVA and WLM, notable adjustments were observed in the estimates of production traits, underscoring the necessity of controlling for DIM in performance analyses. Although age in months showed effect on DMI, RFI, or MY but not significant in this research, the finding may reflect current data structure limitations. With expanded sample sizes, especially at individual level, in future studies, age in months might potentially be identified as a significant factor. This would support more precise age-based classification of animals, facilitating targeted nutritional and management interventions to optimize production efficiency. So, the data derived from on-site production conditions can provide a reliable reference for farm management, after given appropriate analysis. The dynamic relationships between these traits could inform real-time guidance for precision management at various production stages to optimize economic outcomes.

## Data Availability

The raw data supporting the conclusions of this article will be made available by the authors, without undue reservation.
